# Behavioural changes, sharing behaviour and psychological responses after receiving direct-to-consumer genetic test results: a systematic review and meta-analysis

**DOI:** 10.1007/s12687-017-0310-z

**Published:** 2017-06-29

**Authors:** Kelly F. J. Stewart, Anke Wesselius, Maartje A. C. Schreurs, Annemie M. W. J. Schols, Maurice P. Zeegers

**Affiliations:** 1 Department of Complex Genetics and Epidemiology, School of Nutrition, and Translational Research in Metabolism (NUTRIM), Maastricht University Medical Centre, Maastricht, The Netherlands; 2 National Nutrition Surveillance Centre, School of Public Health, Physiotherapy and Sport Science, University College Dublin, Dublin, Ireland; 3 Department of Respiratory Medicine, School of Nutrition and Translational Research in Metabolism (NUTRIM), Maastricht University Medical Centre Maastricht, Maastricht, The Netherlands; 4 CAPHRI School for Public Health and Primary Care, Maastricht University Medical Centre, Maastricht, The Netherlands

**Keywords:** Direct-to-consumer genetic testing, Behaviour change, Psychological responses, Sharing behaviour, Systematic review, Meta-analysis

## Abstract

It has been hypothesised that direct-to-consumer genetic tests (DTC-GTs) could stimulate health behaviour change. However, genetic testing may also lead to anxiety and distress or unnecessarily burden the health care system. The aim is to review and meta-analyse the effects of DTC-GT on (1) behaviour change, (2) psychological response and (3) medical consumption. A systematic literature search was performed in three databases, using “direct-to-consumer genetic testing” as a key search term. Random effects meta-analyses were performed when at least two comparable outcomes were available. After selection, 19 articles were included involving 11 unique studies. Seven studies involved actual consumers who paid the retail price, whereas four included participants who received free genetic testing as part of a research trial (non-actual consumers). In meta-analysis, 23% had a positive lifestyle change. More specifically, improved dietary and exercise practices were both reported by 12%, whereas 19% quit smoking. Seven percent of participants had subsequent preventive checks. Thirty-three percent shared their results with any health care professional and 50% with family and/or friends. Sub-analyses show that behaviour change was more prevalent among non-actual consumers, whereas sharing was more prevalent among actual consumers. Results on psychological responses showed that anxiety, distress and worry were low or absent and that the effect faded with time. DTC-GT has potential to be effective as a health intervention, but the right audience needs to be addressed with tailored follow-up. Research is needed to identify consumers who do and do not change behaviour or experience adverse psychological responses.

**Electronic supplementary material:**

The online version of this article (doi:10.1007/s12687-017-0310-z) contains supplementary material, which is available to authorized users.

## Introduction

The momentum for direct-to-consumer genetic test (DTC-GT) for common disease risks is still increasing among scientists, policy makers, media and the lay public. This is reflected in ongoing debates about what should be offered, to and by whom, and if and how it should be regulated; all with the purpose of finding the best balance between protection of customers and supporting their autonomy (Rafiq et al. 2015; Vayena 2015).

A key argument in this debate is one of the preventive values or clinical utilities. It has been hypothesised that DTC-GTs can serve as tools to stimulate health behaviour change (Bloss et al. 2011a). Primarily, the assumption is that knowledge of an increased disease risk could lead to risk-reducing behaviour among individual customers, as found with penetrant gene testing (Vernarelli et al. 2010). In addition, personal disease risk information can be used to further tailor lifestyle interventions and target the right populations for both interventions and health monitoring, likely resulting in increased effectiveness and cost reduction. However, to date, studies have shown that behaviour change as a result of genetic testing is not always found on the population level (Bloss et al. 2013; Hollands et al. 2016; Smerecnik et al. 2012) or that results remain modest (Covolo et al. 2015; Egglestone et al. 2013), with some subgroups even showing an unhealthy behavioural response to low disease risks (Covolo et al. 2015; Kaufman et al. 2012). In addition, it has been argued that genetic testing could lead to increased anxiety and distress among customers (Lippi et al. 2011), but this has largely been refuted (Nordgren 2014). Moreover, concerns have been raised about the unnecessarily burdening of the already strained health care system (McGuire and Burke 2008). Because genetic information and risk information are difficult to understand (Fausset 2012; McBride et al. 2010), customers may require additional help in interpreting their results correctly if the information provided by testing companies is insufficient. Consumers may then seek help from their health care professional to make sense of their results and to follow up with additional testing.

In order to facilitate evidence-based decision-making with regard to implementation of DTC-GT services and the use thereof for clinical purposes, a quantitative summary of the available data is required. To date, this has not yet been performed. Therefore, the aim of the current study is to review and, where possible, meta-analyse the effects of DTC-GT among a general population on (1) health-related behaviour change, (2) psychological responses and (3) medical consumption, as studied in interventional, cohort, case-control or cross-sectional studies.

## Methods

This study was registered in PROSPERO under registration number CRD42016037927.

### Search strategy

A systematic literature search was performed until January 2017 in three databases: Web of Science, PubMed, and Embase, using “Direct-to-consumer genetic testing” and “Personal genetic testing” as key search terms. In addition, reference lists of and citations to key publications were investigated for eligible studies. No restriction on language or publication date was applied.

### Study selection

First selection was based on title and abstract, after which full texts were reviewed. Studies were included when they researched health behaviour change, psychological responses or medical consumption as a result of genetic disease risk testing delivered directly to consumers. Original studies, including trials and longitudinal and cross-sectional studies, were included while case studies or commentaries were excluded. Studies were restricted to those studying a general population of adults. Because of the focus on multiplex genetic testing, nutrigenetic, pharmacogenetic or sports-related genetic tests were excluded, as well as genetic testing for highly penetrant genes and prenatal or neonatal genetic tests. Both actual consumers, meaning people having purchased the test by themselves, and non-actual consumers, meaning people having obtained the test through a scientific study, were included. Hypothetical consumers, meaning people receiving mock test results, were excluded.

### Data extraction

All data were extracted by KS and checked for consistency by MS. Disagreement was solved through discussion until consensus was reached. Extracted data included the following: author, year, title, study design, country of authors, country of study participants, study name (if applicable), actual or non-actual consumer, cost of test, follow-up duration, total participants and per outcome: the outcome itself (e.g. healthier diet) and the size and unit of the outcome (e.g. 13% changed). Not one specific outcome measure was preferred; acceptable outcome measures included percentages and scores on a validated measurement scale. All data were extracted and cross-checked for comparability.

### Selection of data for meta-analysis

Extracted data were cross-checked for outcomes that were suitable to be combined in meta-analysis, i.e. when outcomes reported the same or similar behaviours with the same unit of measurement. In case of possible overlap between estimates, only the most comprehensive outcome was included for that particular meta-analysis. For example, the percentage of people sharing results with “any health care professional (HCP)” was selected rather than both estimates on “genetic specialist” and “general practitioner” separately in the overall analysis on sharing behaviour. If one study reported more than one outcome, each estimate was included separately in the relevant meta-analysis.

### Data analysis

Due to the relatively young field of research, it was decided that meta-analysis was acceptable when a minimum of two estimates were available for a comparable outcome parameter. A random-effects meta-analysis was performed using the metaprop command of STATA V14.0 software (Freeman and Tukey 1950; Nyaga et al. 2014), and confidence intervals were truncated at 0 or 100%, respectively. Heterogeneity was assessed using the *I* ^2^ statistic (Higgins et al. 2003) and explored by doing sub-analyses on whether the test was offered to the participant free or paid and on whether it involved actual consumers or not. Publication bias was evaluated with Begg’s adjusted rank correlation test (Begg and Mazumdar 1994) and Egger’s regression asymmetry test (Egger et al. 1997).

## Results

### Literature search

**Fig. 1:**
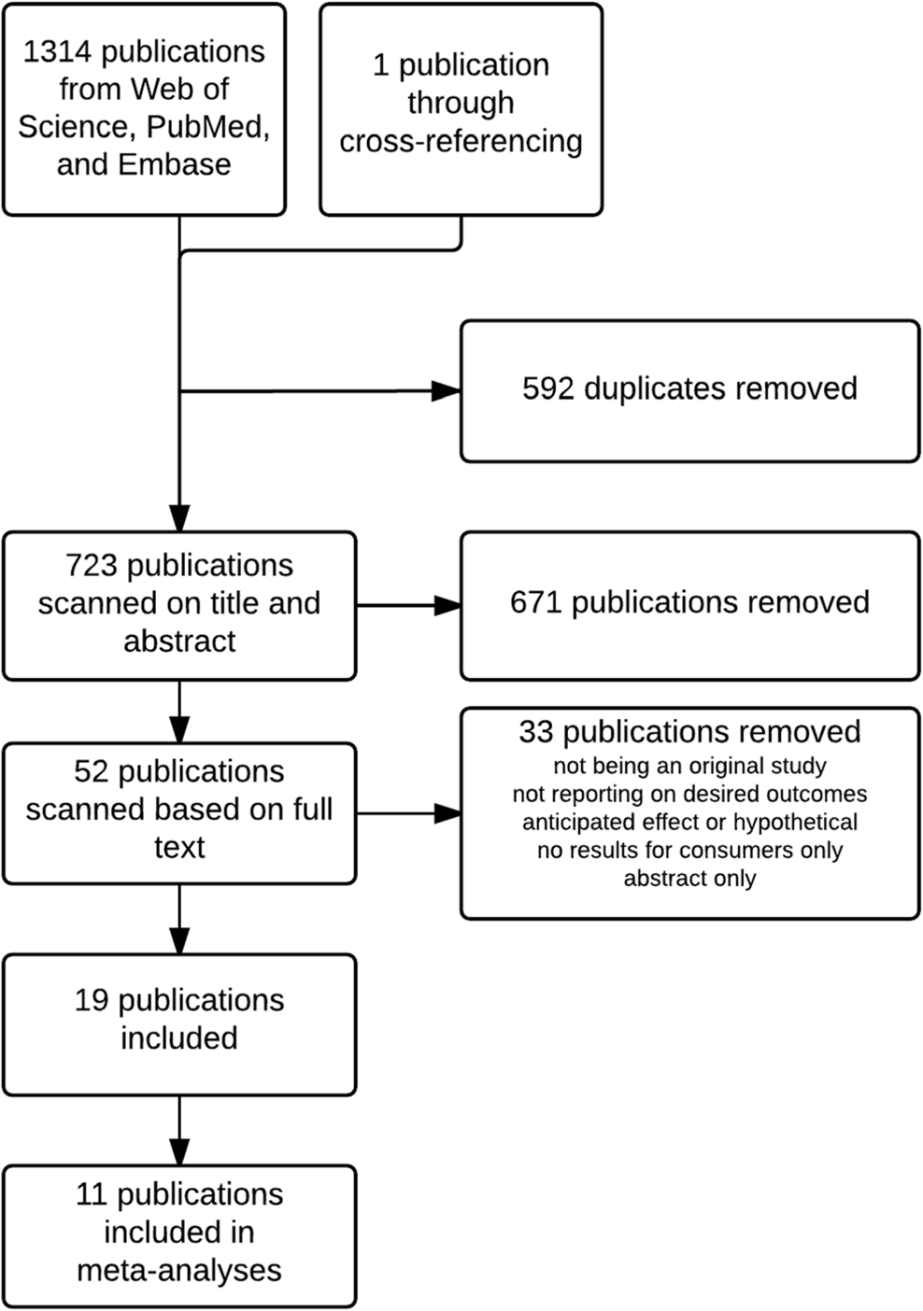
Flow chart of literature search

A total of 1315 publications were identified from the database search and through cross-referencing. After removing 592 duplicates, 723 publications remained for first selection based on title and abstract. Of these, 671 publications were removed based on title and abstract, leaving 52 publications. Of these, 19 publications were included based on full text. Reasons for exclusion were not being an original study, not reporting on desired outcomes, anticipated effect or hypothetical scenarios, no results reported for consumers only or being an abstract only. The literature search is depicted in the flow chart in Fig. 1.

### Study characteristics

The characteristics of the 19 articles, involving 11 unique studies (see Table 1, column 9 for details), included in the systematic review can be found in Table 1. Six studies had a cross-sectional design (Egglestone et al. 2013; Gordon et al. 2012; Kaufman et al. 2012; Lee et al. 2013; McGrath et al. 2016; McGuire et al. 2009), three were longitudinal observational studies (Bloss et al. 2011b, 2013; Boeldt et al. 2015; Carere et al. 2016; Darst et al. 2013, 2014; Kaphingst et al. 2012; O’Neill et al. 2015; Reid et al. 2012; van der Wouden et al. 2016) and two were intervention studies (Haga et al. 2014; James et al. 2011). The number of participants ranged from 60 to 2037, totalling 6672 unique participants. Six unique study populations (Carere et al. 2016; Egglestone et al. 2013; Kaufman et al. 2012; Lee et al. 2013; McGrath et al. 2016; McGuire et al. 2009; van der Wouden et al. 2016) involved actual consumers who paid the full or a reduced retail price for the product. The remaining five studies (Bloss et al. 2011b, 2013; Boeldt et al. 2015; Darst et al. 2013, 2014; Gordon et al. 2012; Haga et al. 2014; James et al. 2011; Kaphingst et al. 2012; Reid et al. 2012) included participants who were offered genetic testing for free as part of a research trial (henceforth referred to as non-actual consumers). In general, the studies involved mostly Caucasian individuals with relatively higher education and income levels. Six studies did not alter the standard procedures of the genetic testing companies and offered no additional counselling as part of the study (Carere et al. 2016; Egglestone et al. 2013; Kaufman et al. 2012; Lee et al. 2013; McGrath et al. 2016; McGuire et al. 2009; Olfson et al. 2016; van der Wouden et al. 2016). Specific details of what the standard procedures entailed were not clearly provided but were likely to have included an online report with online educational materials. All studies had online reports, except for one study arm of Haga et al. (2014) in which the online report was printed and communicated in person by a genetic counsellor. Four studies included counselling from a HCP (genetic counsellor or physician) to explain the results either at delivery (Haga et al. 2014) or after the participant had viewed their results privately (Bloss et al. 2011b, 2013; Boeldt et al. 2015; Darst et al. 2013, 2014; James et al. 2011; Kaphingst et al. 2012; O’Neill et al. 2015; Reid et al. 2012). One study specifically mentioned to have offered additional online education (Gordon et al. 2012). Follow-up durations ranged from 1 week to 1 year, and four studies (Egglestone et al. 2013; Lee et al. 2013; McGrath et al. 2016; McGuire et al. 2009) interviewed all participants at one moment, resulting in different follow-up durations per participant.

**Table 1:** Characteristics of the included studies

Author (year), larger project if applicable	Country of study (country of participants)	Study design	Number of participants	Customer type/price paid	FU duration	Demographic characteristics of the study population	Post-test contact	The same study population as	Notes
Bloss et al. (2011b), SGHI	USA	Longitudinal observational study	2037	Not real/reduced RRP	3 months	55.3% female Mean age (range) = 47 (19–85) 84.2% White Median education level category: some postgraduate education Median income category = $100,000–$149,000	Online report Pro-active genetic counselling outreach by Navigenics; initially only for specific subgroups, later all customers	Bloss et al. (2013), Boeldt et al. (2015), Darst (2013) and Darst et al. (2013)	Participants were employees from health and technology companies who were offered the regular Navigenics Health Compass^a^ at a reduced rate
Bloss et al. (2013), SGHI	USA	Longitudinal observational study	1325	Not real/reduced RRP	1 year	60.2% female Mean age (range) = 48 (19–84) 84.9% Caucasian Median education category: some post-college education Median income category = $100,000–$149,000	See Bloss et al. (2011b)	Bloss et al. (2011b), Boeldt et al. (2015) and Darst et al. (2013)	
Boeldt et al. (2015), SGHI	USA	Longitudinal observational study	2037	Not real/reduced RRP	3 months	55% female Mean age (range) = 47 (19–85) 84% Caucasian Median education category: some post-college education Median income category = $100,000–$149,000	See Bloss et al. (2011b)	Bloss et al. (2011b, 2013)) and Darst et al. (2013)	
Carere et al. (2016), Pgen study	USA	Longitudinal observational study	998	Real/full RRP	6 months	59.9% female Mean age (range) = 47 (19–94) 85.8% White Predominant education category: Some graduate school (36.0%) Predominant income category = <100,000 (56.0%)	Standard procedures of genetic testing companies	van der Wouden et al. (2016)and Olfson et al. (2016)	New customers of 23andMe^a^ and Pathway genomics^a^
Darst et al. (2013), SGHI	USA	Longitudinal observational study	1325	Not real/reduced RRP	14 months	55.6% female Mean age (range) = 51 (23–75) 85.6% Caucasian Modal education category: master’s degree (25%) Median income category = $150,000–$199,999 (15%)	See Bloss et al. (2011a, b)	Bloss et al. (2011a, 2013), Boeldt et al. (2015)and Darst et al. (2014)	
Darst et al. (2014), SGHI	USA	Longitudinal observational study	2024	Not real/reduced RRP	6 months	*Sharers*: 57% female Mean age (range) = 50 (20–85) 84.6% Caucasian Median education category: some post-college Median income category = $150,000–$199,999 *Non-sharers*: 54.6% female Mean age (range) = 45 (19–81) 84.0% Caucasian Median education category: some post-college Median income category = $100,000–$149,999	See Bloss et al. (2011a)	Bloss et al. (2011b,, 2013), Boeldt et al. (2015)and Darst et al. (2013)	
Egglestone et al. (2013)	USA (71.8%), UK (9.4%), Canada (6.6%), Australia (3.9%), other (8.3%)	Cross-sectional study	189	Real/full RRP	Different per participant (no range reported)	37% female Predominant age category = 30–44 (58%) 84.2% Caucasian Predominant education category: postgraduate degree (55.3%)	Standard procedures of genetic testing companies	None	Data included in our study is restricted to actual consumers (excluding potential consumers, as reported) Participants included customers of 9 different DTC-GT companies^a^
Gordon et al. (2012), CPMC	USA	Qualitative cross-sectional study	60	Not real/free	At least 3 months	60% female Average age = 48.9 68% Caucasian Predominant education category: college degree or more (60%)	Online report, with additional educational material offered online and in educational sessions	None	Data included in our study is the quantitative data reported by the authors The genetic testing is offered through the study for coronary artery disease, type 2 diabetes, haemochromatosis, prostate cancer, melanoma, age-related macular degeneration, and lupus. Other non-genetic factors are included to calculate risk estimates
Haga et al. (2014)	USA	Randomised intervention study	300	Not real/free	1 week	27% female Predominant age category = 18–29 (44%) 68% White Predominant education category: bachelor’s degree or higher (72%)	Online only or printed and communicated in person, depending on randomised condition	None	Non-diabetic participants from the general public were randomized to receive their type 2 diabetes mellitus genetic testing results in person from a certified genetic counsellor or access them online through a secure website. Testing was done through deCODE^a^
James et al. (2011)	USA	Randomised intervention study	150	Not real/free	1 week and 1 year	28% female Predominant age category = 60–69 (47%) Predominant education category: graduate or professional school (47%)	Online report prior to planned appointment with physician. Genetic counselling from Navigenics had been offered, but none of the participants had requested this	None	Participants were recruited from a prevention clinic and received a free modified version of standard test of Navigenics^a^, including only “actionable” diseases (abdominal aneurysm, atrial fibrillation, breast cancer (women only), celiac disease, colon cancer, type 2 diabetes mellitus, Graves’ disease, myocardial infarction, lung cancer, obesity, osteoarthritis and prostate cancer (men only)). The intervention group received the genetic testing in addition to their usual care preventive medicine appointment. They were granted access to the result 1 week before their scheduled preventive medicine appointment. The control group received usual care only, including a wide range of examinations based on medical and family history, physiological examinations and screening tests
Kaphingst et al. (2012), MI	USA	Longitudinal observational study	199	Not real/free	3 months	57% female Mean age (SD) = 35 (4.2) 62% White Predominant education category: college degree or higher (52%)	Mailed report. Participants were contacted within 10 days by a research educator who further explained results, and participants could ask questions	Reid (2012) and O’Neill (2015)	Participants, selected from a large health maintenance organization, received free health screening for 8 common health conditions (diabetes, osteoporosis, heart disease, colon cancer, high cholesterol, lung cancer, high blood pressure and skin cancer)
Kaufman et al. (2012)	USA	Cross-sectional study	1046	Real/full RRP	2–7 months	46% female Predominant age category = 55–74 (42%) 87% White Predominant education category: postgraduate (54%) Predominant income category = >$125,000 (45%)	Standard procedures of genetic testing companies	None	Includes participants from three genetic testing companies (Navigenics^a^, 23andMe^a^ and deCODEme^a^), approached through email
Lee et al. (2013)	NR	Cross-sectional study	80	Real/full RRP	Different per participant (no range reported)	50.0% female Mean age (range) = 44 (23–72) 80.3% White Predominant education category = 4-year college graduate (33.3%) Predominant household income category = <$50.000 (31.6%)	Standard procedures of genetic testing companies	None	Online survey among 23andMe^a^ customers, administered following an in-depth interview. Information about the study was published in the blog that is emailed directly to 23andMe customers
McGrath et al. (2016)	USA	Cross-sectional study	122	Real/full RRP	Not reported	32.8% female Mean age (range) = 34 (19–71) Predominant education category: master’s degree or higher (38.5%) Median income = $90,000	Standard procedures of genetic testing companies	None	Online survey administered to 23andMe^a^ customers
McGuire et al. (2009)	USA	Cross-sectional study	63	Real/full RRP	Different per participant (no range reported)	59% female Predominant age category = 25–34 (38%) 77% Caucasian Predominant education category: bachelor’s degree (36%)	Standard procedures of genetic testing companies	None	Survey administered to general population. Data included is only on participants who “did use” personal genetic testing^a^
Olfson et al. (2016), Pgen	USA	Longitudinal prospective cohort study	1464	Real/full RRP	6 months	61% female Mean age (range) = 44 (23–72) 90% White College degree or more advanced education = 78% Mean household income category = $70,000–$99,999	Carere et al. (2016)	Carere et al. (2016)and van der Wouden et al. (2016)	
O’Neill et al. (2015), MI	USA	Longitudinal observational study	228	Not real/free	10 days	56.6% female Mean age (SD) = 35 (4.2) 62.3% non-Hispanic White Predominant education category: college or more (67.1%)	See Kaphingst et al. (2012)	Kaphingst et al. (2012)and Reid et al. (2012)	
Reid et al. (2012), MI	USA	Longitudinal observational study	1599	Not real/free	Comparing health care use 12 months prior and post testing	59.0% female Predominant age category = 35–40 (61.3%) 61.3% White Predominant education category: college graduate (51.2%)	See Kaphingst et al. (2012)	Kaphingst et al. (2012)and O’Neill et al. (2015)	
van der Wouden et al. (2016), Pgen	USA	Longitudinal prospective cohort study	1026	Real/full RRP	6 months	39.7% female Mean ages = 45–51 85.1% White Predominant education category: some graduate school (35.5%) Predominant income category = $100,000–$199,999 (30.3%)	See Carere et al. (2016)	Carere et al. (2016)and Olfson et al. (2016)	

*CPMC* Coriell Personalized Medicine Collaborative, *FU* follow-up, *MI* multiplex initiative, *NR* not reported, *Pgen* Impact of Personal Genomics study, *RRP* regular retail price, *SGHI* Scripps Genomic Health Initiative

^a^Refers to commercially available services

Seven articles (Bloss et al. 2011b, 2013; Boeldt et al. 2015; Egglestone et al. 2013; Gordon et al. 2012; Kaphingst et al. 2012; Kaufman et al. 2012) reported on behaviour changes, eight articles (Bloss et al. 2011b, 2013; Boeldt et al. 2015; Carere et al. 2016; Egglestone et al. 2013; Haga et al. 2014; James et al. 2011; Kaphingst et al. 2012) on psychological effects and 15 articles (Bloss et al. 2011b, 2013; Boeldt et al. 2015; Carere et al. 2016; Darst et al. 2013, 2014; Egglestone et al. 2013; Gordon et al. 2012; Kaphingst et al. 2012; Kaufman et al. 2012; Lee et al. 2013; McGrath et al. 2016; McGuire et al. 2009; Reid et al. 2012; van der Wouden et al. 2016) on sharing results with others and medical follow-up. Estimates from 11 articles (Bloss et al. 2011b, 2013; Carere et al. 2016; Egglestone et al. 2013; Gordon et al. 2012; Kaphingst et al. 2012; Kaufman et al. 2012; Lee et al. 2013; McGrath et al. 2016; McGuire et al. 2009; van der Wouden et al. 2016) were included in at least one meta-analysis.

### Health-related behaviour change

Table 2 shows the results with regard to behaviour change. A wide range of lifestyle behaviours have been studied, including general dietary practices, fat intake, caffeine intake, vitamin and supplement use, weight loss, alcohol use, smoking and exercise behaviour. All studies reported results as the percentage of people with a certain changed behaviour (Bloss et al. 2011b; Egglestone et al. 2013; Gordon et al. 2012; Kaufman et al. 2012; Olfson et al. 2016), and one study (Bloss et al. 2011b, 2013) also measured actual behaviour through questionnaires. All of these were self-reported. Any positive lifestyle change ranged from 14% for exercise behaviour (Kaufman et al. 2012) to 33% for dietary behaviour (Kaufman et al. 2012) or any lifestyle change (Gordon et al. 2012). Two studies (Bloss et al. 2011b; Olfson et al. 2016) found that between 18 and 22% of pre-test smokers had quit smoking. Between 3.2 and 20.5% of people undergoing genetic testing made a change in their vitamin supplementation (Bloss et al. 2011b; Egglestone et al. 2013; Kaufman et al. 2012). Information-seeking behaviour varied widely, which may also depend on the type of information sought: 1.6% looked up information on their high-risk items (Egglestone et al. 2013), whereas 65% had looked up information about how health habits influenced their risk of disease (Kaphingst et al. 2012). The results from the exercise and dietary fat intake questionnaires showed no change for either outcome compared to pre-testing (Bloss et al. 2011b, 2013). Behaviour change was not mediated by perceived control of the disease or the actual genetic risk that was received (Boeldt et al. 2015).

**Table 2:** Results with regard to behaviour change, psychological responses and medical consumption

Author (year), larger project if applicable	Behaviour change	Psychological responses	Medical consumption
Bloss et al. (2011b), SGHI	*Dietary fat intake* (BDFS): no significant change in dietary fat intake (BL = 16.0 ± 7.9, 3-month FU = 15.2 ± 7.5, *p* = 0.89) *Leisure time exercise* (GLTEQ): no significant change in leisure time exercise level (BL = 28.6 ± 23.0, 3-month FU = 28.6 ± 22.9, *p* = 0.61) *Change in vitamin use* = 20.5% of study population indicated a changed pattern of use (18% increased or started, 2.5% decreased or quit; BL = 82.8% used vitamins) *Change in alcohol use* = 11.2% indicated a changed pattern of use (2.0% increased or started, 9.2% decreased or quit; BL = 80.0% used alcohol) *Change in tobacco use*: of pre-test smokers 17.6% had quit smoking and 21.8% of had decreased their use. Of all participants, 7.6% had increased or started smoking	*State anxiety* (state anxiety subscale of SSTAI, <39 considered low-anxiety state): no significant change in state anxiety at FU (BL = 35.2 ± 9.6, 3-month FU = 34.6 ± 10.0, *p* = 0.80) *Test-related distress* (IES-R, score of >8 and >23 points on avoidance and intrusion subscales indicate “some impact” and “clinically relevant distress”, respectively): No significant TRD (3-month FU = 3.2 ± 7.1, 90.3% scored <8 and 97.2% scored <23)	*Discussing results with Navigenics genetic counsellor* = 10.4% *Sharing with physician or HCP* = 26.5% Sharing was not associated with changes in test-related distress or anxiety level. Only sharing with physician was associated with lower dietary fat intake and increased exercise behaviour at follow-up
Bloss et al. (2013), SGHI	*Dietary fat intake* (BDFS): no significant change in dietary fat intake (BL = 15.9 ± 7.8, 1-year FU = 14.8 ± 7.3, *p* = 0.34) *Leisure time exercise* (GLTEQ): no significant change in leisure time exercise level (BL = 27.9 ± 23.1, 1-year FU = 29.8 ± 24.4, *p* = 0.39)	*State anxiety* (state anxiety subscale of SSTAI, <39 considered low-anxiety state): no significant change in state anxiety at FU (BL = 35.0 ± 9.5, 3-month FU = 34.2 ± 10.0, *p* = 0.50) *Test-related distress* (IES-R), score of >8 and >23 points on avoidance and intrusion subscales indicate “some impact” and “clinically relevant distress”, respectively): No significant TRD, significant decrease in TRD between 3-month and 1-year FU (*p* = 0.03) (1-year FU = 1.2 ± 3.4, 96.8% scored <8 and 99.7% scored <23)	*Discussing results with Navigenics genetic counsellor* = 14.1% *Sharing with physician or HCP* = 39.5% Discussing results with a Navigenics genetic counsellor was associated with higher anxiety, TRD and actual screening test completion, but not with fat intake, exercise behaviour or the intention to complete screening tests with greater frequency Sharing results with a physician or HCP was associated with higher exercise, actual screening test completion and intention to increase frequency of screening, but not with anxiety, TRD or fat intake. *Change in health screening behaviours*: the mean number of screening tests completed since receiving test results was 5.3 ± 2.8 at 1-year FU. There was no significant difference with the mean at 3-month FU (*p* = 0.43)
Boeldt et al. (2015), SGHI	*Dietary fat intake* (BDFS) and *leisure time exercise* (GLTEQ): no significant relationships between perceived control or genetic risk and fat intake or leisure time exercise at 3-month FU	*Test-related distress* (IES-R): higher genetic risk and low perceived control associated with higher distress *Anxiety*: participants receiving a high genetic risk and perceiving their most feared disease as controllable via lifestyle changes, experienced the lowest levels of anxiety at 3-month FU	*Sharing with physician or HCP*: higher genetic risk associated with greater likelihood of sharing with physician *Change in self-check for breast cancer*: higher perceived control and high genetic risk associated with increased likelihood of self-checking
Carere et al. (2016), Pgen study		*Anxiety* (GAD-2): positive GAD-2 screen for anxiety/panic disorder at 6-month FU = 14.5% (BL = 15.8%; *p* = 0.33) *Decision regret* (score 0–100) = 58.4% reported no decision regret, 97.4% reported ≤40/100	*Consultation with HCP after testing* = 34.9%
Darst et al. (2013), SGHI			*Reasons for using GC*: most mentioned reasons were taking advantage of a free service, wanting more information on risk calculations, and because they were contacted by the GC. The least commonly reported reason was a perceived lack of understanding of one’s results. *Reasons for not using GC*: most mentioned reason was feeling to already understand the results. *Outcomes of consulting a GC*: improved understanding of their own results and feeling more educated about genetics in general. For 30.4% consulting a GC made them more likely to discuss their results with their physician
Darst et al. (2014), SGHI			*Demographic characteristics of subjects who shared with physician or HCP*: sharers were older (50.0 ± 12.5 vs. 45.4 ± 11.6; *p* = <0.001), had a higher annual income (150,000–199,000 vs. 100,000–149,000; *p* = 0.1), more likely to be married (73.2 vs. 68%; *p* = 0.005) and more likely to identify with a religion (80.5 vs. 74.2%; *p* = 0.004). *Behavioural characteristics of subjects who shared with physician or HCP*: sharers reported a greater amount of exercise per week (GLTEQ; 27.0 ± 24.9 vs. 22.3 ± 23.3; *p* = 0.003), lower dietary fat intake (BDFS; 15.0 ± 8.1 vs. 16.0 ± 7.8; *p* = 0.02) and more frequent physician visits per year (3.8 vs. 3.3; *p* = <0.001) *Other characteristics of subjects who shared with physician or HCP*: fewer sharers reported overall concerns related to testing (44.6 vs. 52.7% yes; *p* = 0.001) and privacy issues about the data (33.2 vs. 38.5% yes; *p* = 0.03). More sharers reported to greatly value risk information (78.2 vs. 68.8%)
Egglestone et al. (2013)^a^	*Any change in health behaviour due to test results* = 27.3% *Healthier diet* = 12.7% *Stopped or reduced caffeine intake* = 1.6% *Taking vitamins or supplements* = 3.2% *More exercise* = 6.3% *Lost weight* = 1.6% *Other* (*e.g. wearing sunglasses*) = 3.7% *Generally reducing risk conditions* = 1.1% *Looking into high risk items* (*either research, talking with doctor or medical tests*) = 1.6% *Preventative checks such as eye tests* = 2.1%	*Any change in health anxiety due to test results* = 23.8% *Decreased health anxiety due to test results* (out of total *n*) = 15% *Increased health anxiety due to test results* (out of total *n*) = 1.6% *General health anxiety*: no significant difference in anxiety between consumers and potential consumers (3.80 vs. 4.22; *p* = 0.63) *Anxiety about developing a serious disease*: no significant difference in anxiety between consumers and potential consumers (3.52 vs. 3.92; *p* = 0.30)	*Preventative checks such as eye tests* = 2.1%
Gordon et al. (2012), CPMC	*Change in lifestyle* = 33%		*Shared results with a HCP* = 42% (additional 23% intended to share but had not yet done so) *Characteristics of subjects who shared with physician or HCP*: more older than younger participants shared (53 vs. 29%) *Reasons for sharing*: most mentioned reason was so the provider could take some action or offer advice to reduce risk. Only 1 participant (1.6%) shared to ask the HCP to help interpret the results
Haga et al. (2014)		*Distress* (MICRA, range 0–30): very low levels of distress at 1-week FU (mean 2.27). Distress scores were not different between participants with and without an increased risk for T2DM. *Anxiety and depression* (IPQ-R emotional representations subscale, range 6–30): low levels based on T2DM results (mean 11.9)	
James et al. (2011)		*Worry*: no significant differences between intervention and control group in percentages of participants reporting being somewhat or very worried about developing a number of tested diseases at 1-week FU and 1-yeaR FU (range = 40–70%)	
Kaphingst et al. (2012), MI	Measured at 3-month FU: *Information seeking about the effect of personal health habits on risk of health conditions* = 65% *Information seeking about the effect of family history on risk of health conditions* = 36%	Measured on a scale of 1 (not at all)–7 (great deal) at 10-day FU: *Nervous* = 2.6 (1.7) *Afraid* = 1.8 (1.5) *Confused* = 1.7 (1.3) *Regretful* = 1.3 (0.9) Reactions were not associated with the number of variants with increased risk carried	Measured at 3-month FU: *Discussed test results with someone* = 77% *Discussed test results with a HCP* = 1% *Discussed results with family* = 18% *Discussed results with spouse* = 20%
Kaufman et al. (2012)	*More careful about diet after viewing results* = 33.3% (participants with a positive family history were more likely to be more careful about their diet (*p* = 0.03)) *Changed at least 1 medication or supplement regimen* = 16% *Changed a dietary supplement* = 10% (participants with poorer self-perceived health were more likely to change their supplement regimen (*p* = 0.007)) *Exercising more* = 14% *Sought additional information about at least 1 health condition covered in their test* = 43%		*Shared results with at least 1 HCP* = 28% *Shared results with GP* = 20% *Shared results with genetic counsellor* = 1% *Shared results with other HCP* = 19% *Shared results with more than 1 provider* = 11% *Characteristics of sharers with HCP*: participants reporting getting regular physical exams and those with reported poorer self-perceived health were significantly more likely to share with a HCP (*p* = 0.001 and *p* = 0.03, respectively). *Result of sharing*: people who shared with their HCP were significantly more likely to get a follow-up test, change a prescription or supplement regimen and be more careful about their diet. Of the people who shared with a HCP, 49% said they learned something new and useful from their results compared to 27% among non-sharers (*p* < 0.0001). *Follow-up with additional laboratory tests as a result of receiving their data* = 10% (26% of people who shared with a HCP compared to 2% of non-sharers; participants with a positive family history were more likely to have gotten follow-up tests (*p* = 0.001))
Lee et al. (2013)			*Shared results with a HCP* = 56.6% *Shared results with family* = 98.3% *Shared results with friends* = 81.7% *Sharing results online* (23andMe website, Facebook, other platforms) = 66.7% *Sources consulted for help*: mostly internet websites (70.4%) and 23andMe Help (53.7%), compared to 22.7% from a HCP
McGrath et al. (2016)			*Shared results with anyone* = 82.2% *Shared results with a medical professional* = 10.7% *Shared results with family* = 40.9% *Shared results with spouse* = 38.5%
McGuire et al. (2009)			*Shared results with physician* = 53%
Olfson et al. (2016), Pgen	*Quit smoking* = 22% (of current smokers) *Started smoking* = 1% (of never and former smokers)		
O’Neill et al. (2015), MI		*Psychological responses to testing* *Neutral* = 53.9% (most frequently were “not surprised” at 14.2% and “surprised” at 10.8%) *Positive* = 26.9% (most common was “interesting” at 9.2%) *Negative* = 19.2% (most common was “nervous” at 6.5%) No significant differences by gender, race and education were found with regard to frequency of reported responses within the three categories	
Reid et al. (2012), MI			Difference 12 months prior to and 12 months post testing, comparing people who completed genetic testing and those who were lost or excluded before testing: *Visited HCP* (*% of participants*): no significant difference *Number of physician visits*: no significant difference Difference 12 months prior to and 12 months post testing, comparing people who completed genetic testing and those who were lost or excluded before testing: *Laboratory tests or procedures* (*% of participants*): no significant difference
van der Wouden et al. (2016), Pgen			*Discussed results with at least 1 HCP* = 34.7% *Discussed results with GP* = 27.1% *Discussed results with genetic specialist* = 1% *Characteristics of sharers*: participants who discussed with a HCP were more frequently women, did not have a college degree (for GP only), were parents and had a positive screen for baseline anxiety

*BDFS* Block Dietary Fat Screener, *GAD-2* two-item Generalized Anxiety Disorder screener, *GLTEQ* Godin Leisure-Time Exercise Questionnaire, *IES-R* Impact of Events Scale-Revised, *IPQ-R* Illness Perception Questionnaire Revised, *MICRA* Multidimensional Impact of Cancer Risk Assessment, *SSTAI* Spielberger State–Trait Anxiety Inventory, *T2DM* type 2 diabetes mellitus, *TRD* test-related distress, *FU* follow-up, *BL* baseline

^a^Some values have been recalculated with “actual consumers” only (*n* = 189)

### Meta-analyses for health-related behaviour change

Outcome parameters that were considered suitable for meta-analysis were the percentages of people with (1) any positive lifestyle change, (2) improved dietary practices, (3) improved exercise practices, (4) quitting smoking, (5) changed supplementation use and (6) information-seeking behaviours. The results are displayed in Table 3, and forest plots can be found on Online resource 1. The overall proportion of people showing any positive lifestyle change after DTC-GT was 24% (95% CI 15–34). For this estimate, no publication bias was detected with either Begg’s (*P* = 0.573) or Egger’s test (*p* = 0.571). More specifically, improved dietary practices was reported by 16% (95% CI 0–38), improved exercise practices also by 12% (95% CI 10–14), quitting smoking by 19% of pre-test smokers (95% CI 13–25) and change in supplementation use by 11% (95% CI 2–21). More than a third of participants (36%, 95% CI 7–66) had sought information, including information-seeking behaviour for the disease itself, healthier lifestyle and heritability.

**Table 3:** Full and subgroup meta-analyses on health-related behaviour change

Comparison	No. of estimates included (no. unique studies)	% of people (95% CI)	*I* ^2^%
Any positive lifestyle change	6 (5) (Bloss et al. 2011b; Egglestone et al. 2013; Gordon et al. 2012; Kaufman et al. 2012; Olfson et al. 2016)	24 (15–34)	95.9
Real customers	4 (3) (Egglestone et al. 2013; Kaufman et al. 2012; Olfson et al. 2016)	24 (12–36)	97.5
Not real customers	2 (2) (Gordon et al. 2012; Bloss et al. 2011b)	21 (16–27)	–
Full price	4 (3) (Egglestone et al. 2013; Kaufman et al. 2012; Olfson et al. 2016)	24 (12–36)	97.5
Reduced price	1 (1 Bloss et al. (2011a)	–	–
Free	1 (1) (Gordon et al. 2012)	–	–
Improved dietary practices	3 (2) (Egglestone et al. 2013; Kaufman et al. 2012)	16 (0–38)	99.4
Real customers	3 (2) (Egglestone et al. 2013; Kaufman et al. 2012)	16 (0–38)	99.4
Not real customers	–	–	–
Full price	3 (2) (Egglestone et al. 2013; Kaufman et al. 2012)	16 (0–38)	99.4
Reduced price	–	–	–
Free	–	–	
Improved exercise practices	2 (2) (Egglestone et al. 2013; Kaufman et al. 2012)	12 (10–14)	–
Real customers	2 (2) (Egglestone et al. 2013; Kaufman et al. 2012)	12 (10–14)	–
Not real customers	–	–	–
Full price	2 (2) (Egglestone et al. 2013; Kaufman et al. 2012)	12 (10–14)	–
Reduced price	–	–	–
Free	–	–	–
Quit smoking	2 (2) (Bloss et al. 2011b; Olfson et al. 2016)	19 (13–25)	–
Real customers	1 (1) (Olfson et al. 2016)	–	–
Not real customers	1 (1) (Bloss et al. 2011b)	–	–
Full price	1 (1) (Olfson et al. 2016)	–	–
Reduced price	1 (1) (Bloss et al. 2011b)	–	–
Free	–	–	–
Change in supplement use	3 (3) (Bloss et al. 2011b; Egglestone et al. 2013; Kaufman et al. 2012)	11 (2–21)	98.6
Real customers	2 (2) (Egglestone et al. 2013; Kaufman et al. 2012)	8 (6–9)	–
Not real customers	1 (1) (Bloss et al. 2011b)	–	–
Full price	2 (2) (Egglestone et al. 2013; Kaufman et al. 2012)	8 (6–9)	–
Reduced price	1 (1) (Bloss et al. 2011b)	–	–
Free	–	–	–
Information-seeking behaviour	4 (3) (Egglestone et al. 2013; Kaphingst et al. 2012; Kaufman et al. 2012)	36 (7–66)	99.6
Real customers	2 (2) (Egglestone et al. 2013; Kaufman et al. 2012)	12 (11–14)	–
Not real customers	2 (1) (Kaphingst et al. 2012)	51 (46–55)	–
Full price	2 (2) (Egglestone et al. 2013; Kaufman et al. 2012)	12 (11–14)	–
Reduced price	–	–	–
Free	2 (1) (Kaphingst et al. 2012)	51 (46–55)	–

– = not calculated or not available

*CI* confidence interval

Results of the subgroup analyses show similar levels of any positive lifestyle change among actual consumers and participants recruited through trials (24 and 21%, respectively). Information-seeking behaviour was more prevalent among participants recruited in trials receiving a free test (51%, compared to 12% of actual consumers paying the full price). Subgroup analyses on the other outcomes were not possible. It should be noted that only one study of non-actual consumers reported on positive lifestyle changes.

### Psychological responses

Table 2 shows the results of all findings with regard to psychological responses. In general, the effect of testing on a number of psychological responses, including anxiety, distress and worry, was low or absent (Bloss et al. 2011b, 2013; Carere et al. 2016; Egglestone et al. 2013; Haga et al. 2014; Kaphingst et al. 2012; O’Neill et al. 2015) and this effect faded with time (Bloss et al. 2013). Increased perceived control led to lower levels of anxiety and distress in one study (Boeldt et al. 2015). One study showed higher levels of distress with increased genetic risk (Boeldt et al. 2015), whereas another study found no difference (Haga et al. 2014). Two studies that reported on regret of testing (Carere et al. 2016; Kaphingst et al. 2012) found low levels of regret. Gender, race and education were not associated with frequency of reported positive, neutral or negative responses (O’Neill et al. 2015).

The outcome parameters between the studies were considered too heterogeneous for meta-analysis, so no meta-analysis was performed.

### Medical consumption

Table 2 shows the results of all findings with regard to any form of sharing behaviour and additional medical follow-up. Most people shared their results with someone, which could be family and friends, online or a HCP. In general, more people shared with their HCP (general practitioner or other) than with a genetic specialist. The percentage of participants discussing the results with family or friends ranged widely between the included studies, from around 20% in one (Kaphingst et al. 2012) to 98% in another (Lee et al. 2013).

The effect of DTC-GT on preventive screening behaviour was little to none (Bloss et al. 2013; Egglestone et al. 2013), although greater likelihood of self-checking was found with higher perceived control of the disease (through lifestyle changes or medical attention) and higher genetic risk received (Boeldt et al. 2015). However, sharing with a HCP led to additional follow-up tests (Kaufman et al. 2012) and increased screening behaviour (Bloss et al. 2013). One study (Reid et al. 2012) specifically researched the effect of DTC-GT on medical follow-up comparing to a control group and found no difference in the percentage of people who visited their HCP, the number of visits and the percentage of people with lab tests or procedures.

### Result of sharing

People who had shared their results with a HCP had lower dietary fat intake and higher exercise at follow-up (Bloss et al. 2011b, 2013), were more careful about their diet and more often changed a prescription or supplemental regimen (Kaufman et al. 2012). Sharing with a HCP did not lead to lower levels of anxiety or distress in one study at 3 months post testing (Bloss et al. 2011b), while sharing with a genetic specialist actually increased anxiety and distress at 1 year post testing in the same study (Bloss et al. 2013). More HCP sharers than non-sharers indicated that they had learned something new and useful from their genetic tests (Kaufman et al. 2012). Sharing with a genetic specialist improved participant’s understanding of own results and made them feel more educated about genetics (Darst et al. 2013). About a third of the participants in the same study reported that sharing with a genetic specialist made them more likely to discuss their results with their physician (Darst et al. 2013).

Five studies researched characteristics of people who shared their results with a HCP (Boeldt et al. 2015; Darst et al. 2014; Gordon et al. 2012; Kaufman et al. 2012; van der Wouden et al. 2016). In summary, people who shared their results with a HCP were more likely to be women (van der Wouden et al. 2016), older (Darst et al. 2014; Gordon et al. 2012), married (Darst et al. 2014) or parents (van der Wouden et al. 2016). They also appeared to have a higher annual income (Darst et al. 2014; van der Wouden et al. 2016) and identified with a religion (Darst et al. 2014). They exercised more and had a lower fat intake (Darst et al. 2014), more frequently visited their physician (Darst et al. 2014; Kaufman et al. 2012), had poorer self-perceived health (Kaufman et al. 2012) and more often had a positive screen for anxiety prior to testing (van der Wouden et al. 2016). Finally, they also had fewer concerns related to testing and privacy issues regarding the data (Darst et al. 2014), greatly valued the risk information (Darst et al. 2014) and had greater genetic risks (Boeldt et al. 2015).

### Meta-analyses for medical consumption

Outcome parameters that were considered suitable for meta-analysis were the percentages of people who shared their results with (1) any HCP, (2) general practitioners, (3) genetic specialists and (4) family and/or friends and the (5) percentage of people undergoing preventative checks. Table 4 shows the results of these meta-analyses. One third of participants (33%, 95% CI 18–48) reported to have shared their results with any HCP, among which general practitioners, genetic specialist or other specialists. For this estimate, no publication bias was detected with either Begg’s (*P* = 0.458) or Egger’s (*p* = 0.120) test. Of these, 23% (95% CI 21–25) had shared with their general practitioners whereas 5% (95% CI 1–10) had shared with a genetic specialist. Half of participants (50%, 95% CI 14–85) reported to have shared their results with family and/or friends. The percentage of people having preventive checks, including screening practices and laboratory tests, was 7% (95% CI 5–8).

**Table 4:** Full and subgroup meta-analyses on medical consumption

Comparison	No. of estimates included (no. unique studies)	% of people (95% CI)	*I* ^2^%
Sharing with (at least 1) HCP	8 (8) (Bloss et al. 2013; Gordon et al. 2012; Kaphingst et al. 2012; Kaufman et al. 2012; Lee et al. 2013; McGrath et al. 2016; McGuire et al. 2009; van der Wouden et al. 2016)	33 (18–48)	99.4
Real customers	5 (5) (Kaufman et al. 2012; Lee et al. 2013; McGrath et al. 2016; McGuire et al. 2009; van der Wouden et al. 2016)	35 (25–46)	95.9
Not real customers	3 (3) (Bloss et al. 2013; Gordon et al. 2012; Kaphingst et al. 2012)	27 (0–58)	99.7
Full price	5 (5) (Kaufman et al. 2012; Lee et al. 2013; McGrath et al. 2016; McGuire et al. 2009; van der Wouden et al. 2016)	35 (25–46)	95.9
Reduced price	1 (1) (Bloss et al. 2013)	–	–
Free	2 (2) (Gordon et al. 2012; Kaphingst et al. 2012)	1 (0–3)	–
Sharing with general practitioner	2 (2) (Kaufman et al. 2012; van der Wouden et al. 2016)	23 (21–25)	–
Real customers	2 (2) (Kaufman et al. 2012; van der Wouden et al. 2016)	23 (21–25)	–
Not real customers	–	–	–
Full price	2 (2) (Kaufman et al. 2012; van der Wouden et al. 2016)	23 (21–25)	–
Reduced price	–	–	–
Free	–	–	–
Sharing with genetic specialist	3 (3) (Bloss et al. 2013; Kaufman et al. 2012; van der Wouden et al. 2016)	5 (1–10)	98.9
Real customers	2 (2) (Kaufman et al. 2012; van der Wouden et al. 2016)	1 (1–2)	–
Not real customers	1 (1) (Bloss et al. 2013)	–	–
Full price	2 (2) (Kaufman et al. 2012; van der Wouden et al. 2016)	1 (1–2)	–
Reduced price	1 (1) (Bloss et al. 2013)	–	–
Free	–	–	–
Sharing with family and/or friends	6 (3) (Kaphingst et al. 2012; Lee et al. 2013; McGrath et al. 2016)	50 (14–85)	99.6
Real customers	4 (2) (Lee et al. 2013; McGrath et al. 2016)	65 (32–98)	99.0
Not real customers	2 (1) (Kaphingst et al. 2012)	19 (15–23)	–
Full price	4 (2) (Lee et al. 2013; McGrath et al. 2016)	65 (32–98)	99.0
Reduced price	–	–	–
Free	2 (1) (Kaphingst et al. 2012)	19 (15–23)	–
Preventive checks	2 (2) (Egglestone et al. 2013; Kaufman et al. 2012)	7 (5–8)	–
Real customers	2 (2) (Egglestone et al. 2013; Kaufman et al. 2012)	7 (5–8)	–
Not real customers	–	–	–
Full price	2 (2) (Egglestone et al. 2013; Kaufman et al. 2012)	7 (5–8)	–
Reduced price	–	–	–
Free	–	–	–

– = not calculated, available or appropriate

*CI* confidence interval, *HCP* health care professional

Results of the subgroup analyses show higher percentages of participants who shared their results with (at least one) HCP among customers paying the full price versus those receiving it for free (35 vs. 1%) and among actual consumers versus non-actual consumers (35 vs. 27%). Participants had shared more often with family and/or friends among the studies of actual consumers paying full price (65%) compared to studies involving non-actual consumers receiving the test for free (19%). Subgroup analyses for these subgroups on the other outcomes were not possible. It should be noted that both estimates of sharing with family and/or friends come from one study.

## Discussion

### Changes in response to testing

#### Health behaviour change

The results of our meta-analyses show that, when genetic testing is offered directly to consumers without additional lifestyle counselling, the effects on behaviour change are modest, whereas on average, just under a quarter of people reported any health behaviour-related change, little is known about the size of the effect and whether change is maintained at a long term. For example, studies asking participants “whether they changed” found both positive and negative effects, but with more objective measures of behaviour, the finding was not replicated (Bloss et al. 2011b, 2013; Boeldt et al. 2015). Therefore, it is paramount that future studies measure behaviour change pre- and post testing, with validated and more objective measures. Furthermore, although we found that 19% of smokers quit smoking after undergoing DTC-GT, it is unclear if cessation was maintained at follow-up. A meta-analysis on genetic testing-based smoking cessation interventions found a significant improvement in cessation rates at short-term follow-up but not at long-term follow-up (≥6 months) (Smerecnik et al. 2012). Finally, sub-analyses show no great difference in “any positive lifestyle change” between actual consumers paying the full price and non-actual consumers paying a reduced price or receiving the test for free, and therefore, large differences in effect for this outcome in normal practice of DTC-GT services need not be expected.

A possible distortion in the found effects may be due to it being likely that not all participants required change in a specific behaviour, thereby underestimating the relevant impact. In contrast, estimates for quitting smoking were reported for pre-test smokers only, showing how many participants who required changing had changed the behaviour. Because it is unclear how many participants required a specific behaviour change, the effects may be greater when focussing only on those requiring change.

#### Undesired consequences of testing

Typical concerns of opponents of DTC-GT are potential adverse psychological responses, such as anxiety or test-related distress, and inappropriate responses to testing, such as foregoing screening when receiving reduced risks or taking unnecessary or inappropriate preventive measures, including the change of prescription medication. The results of our study show that there is currently little to no evidence for serious adverse psychological responses among consumers. In addition, only a small percentage of people show potentially inappropriate responses, such as changing prescription and over-the-counter medication (Kaufman et al. 2012). However, the genetic testing results in the study in which this was found also included some pharmacogenomic testing, which may explain and possibly justify the medication change by consumers, provided that the pharmacogenomic test results were understood correctly. Similarly, it is not necessarily true that starting a supplementation regimen is a positive lifestyle change (Myung et al. 2010). Although the percentages of people with adverse responses or reactions are low, the consequences when it does happen may still be significant. These are delicate issues, which should receive attention from both genetic testing companies and HCPs.

#### Sharing behaviour

We found that a third of participants shared their results with at least one HCP. This often led to additional follow-up tests or health screening (Bloss et al. 2011b; Kaufman et al. 2012). In some cases, this will result in early detection and prevention (e.g. participant no. 11 in Gordon et al. 2012), whereas in other cases, additional tests may be done merely to reassure the individual, unnecessarily burdening the health care system. It has been estimated that the highest downstream costs may range from $40 to $20.604 (Giovanni et al. 2010).

A significant role remains for genetic testing companies to put great effort in communication of results and aid their customers in correct interpretation of their findings without help of any health care professionals. The study that reported only 1% of people contacting a HCP, compared to the 28–57% reported by other studies, had also contacted all participants by telephone within 10 days of receiving mailed results (Kaphingst et al. 2012). During this call, results were explained further and participants had the chance to ask questions. This approach may have prevented the need to contact a HCP and may offer a suggestion for post-test communication. Other examples of how disease risk information may be communicated effectively is given by Lautenbach et al. (2013).

Many consumers shared with family and/or friends. Particularly sharing with family, due to shared genetics, may also lead to improved lifestyle among individuals who did not undergo testing. However, as effects on the lifestyle of test participants currently prove to be modest, the effect on the non-tested individual is likely to be minimal.

### Different values of DTC-GT

Although it is tempting to base regulatory decisions regarding DTC-GT on its potential to improve health (i.e. preventive value or clinical utility), health should not be the only criterion to judge by. A different but related value of DTC-GT is one of the personal values and autonomy (Chung and Ng 2016; Vayena 2015). Several studies on reasons of purchasing DTC-GT revealed that the main reason is satisfying curiosity of the consumer (Gollust et al. 2012; Ormond et al. 2011; Su et al. 2011), indicating that the entertainment value should receive considerable attention in justifying these tests (Chung and Ng 2016). In addition, it may serve as a source of information for informed health-related decision-making, such as long-term life decisions. The value of genetic testing for individuals who purchase it with the purpose of learning of disease risks to make informed health decisions may be of a very different nature from the value for those purchasing out of mere interest and entertainment. Therefore, self-selection of undergoing testing for health-related reasons may be an important determinant of finding an effect, as a recent meta-analysis (Hollands et al. 2016) found no effect of receiving genetic information on behaviour change in randomised trials. To use DTC-GT as an effective health behaviour intervention, it may need to be combined with continued lifestyle intervention and/or counselling, which will need tailoring to the intentions of the individual. Doing so will principally attract more consumers with the intention of improving health, but will also stimulate those with and without prior intention of improving health in the process of behaviour change. The effects on health behaviour may then be much larger compared to the effects found from genetic testing like most DTC-GT companies currently offer, which may more often be purchased out of mere entertainment.

### Demographics of the study populations

A common criticism is the assumption that most DTC-GT users involve predominantly white individuals with higher income and education level. Kaufman et al. (2012) explicitly compared their study population with the general US population and found that white men with higher incomes and education were indeed overrepresented in their study. Although other studies did no direct comparisons to the general population, most studies support this finding. If DTC-GT services indeed stimulate healthier lifestyles, this finding could further contribute to socio-economic health differences.

### Limitations

The current study carries several limitations, which are partly due to limitations in the primary studies. Firstly, the reported estimates are mostly not compared to a control group and may reflect natural behaviour changes. However, as several studies specifically included “as a result of receiving your report” in their questions, the effect might be mitigated. Secondly, self-reporting of behaviour change and social desirability might have lead to reporting bias and an overestimation of the effect. Therefore, future studies should include more objective forms of behaviour measurements. Thirdly, estimates of the percentage of people with any positive lifestyle change as well as those who shared with any HCP may have been underestimated. This is due to studies reporting percentages for each behaviour or HCP separately, which could not simply be combined into one summary estimate as participants may changed multiple behaviours or shared with multiple HCPs resulting in participants being counted twice. To reduce the effect as much as possible, only the most overall estimate was included. Fourthly, the five publications of the Scripps Genomic Health Initiative (Bloss et al. 2011b, 2013; Boeldt et al. 2015; Darst et al. 2013, 2014) were considered non-actual consumers due to the recruitment through the research project. However, it should be noted that the participants were exposed to the same Navigenics advertising, imagery and information as regular Navigenics customers, in addition to the study-related information. Fifthly, some meta-analyses are based on a small number of studies, and these estimates should therefore be interpreted with caution. Finally, high levels of heterogeneity were found in the meta-analyses, remaining after sub-analyses by actual consumers versus non-actual consumers. Possible explanations may include the specific behaviour changes that were studied and combined (e.g. “healthier diet” vs. “more careful about diet”), differences in post-test contact and follow-up duration of the included studies and different study designs used. Unfortunately, no subgroup analyses were possible on the duration of follow-up due to the wide range of follow-up durations within and between studies. Longer duration of follow-up is likely to reduce the percentage of people who have maintained a changed lifestyle and increase the percentage of people who have shared their results (Bloss et al. 2011b, 2013).

## Conclusion

Although DTC-GT has the potential to be cost-effective as a health intervention, both the genetic testing and subsequent actions (such as post-test counselling or additional lifestyle interventions) will have to be offered in the right way to the right target audience with tailored follow-up. In order to identify and target this population, research is needed on the characteristics of consumers who do and do not change behaviour or experience adverse psychological responses and who need or desire additional medical attention. Only then can DTC-GT be used, other than for its entertainment value, for the population at whole with maximum benefits at minimum costs.

## Electronic supplementary material

ESM 1 (DOCX 2620 kb)
