# Supplementary material for: Behavioural changes, sharing behaviour and psychological responses after receiving direct-to-consumer genetic test results: a systematic review and meta-analysis
Source: J Community Genet. 2017 Jun 29;9(1):1–18. doi: 10.1007/s12687-017-0310-z (PMC5752648; doi:10.1007/s12687-017-0310-z)
Supplement: Supplementary file 1 — (DOCX 2620 kb) [file 12687_2017_310_MOESM1_ESM.docx]

# Online resource 1: Forest plots of all meta-analyses

Belonging to:

Behavioural changes, sharing behaviour and psychological responses after receiving direct-to-consumer genetic test results: a systematic review and meta-analysis

Journal of Community genetics

Kelly F. J. Stewart^A*^, Anke Wesselius^A^, Maartje A. C. Schreurs^B^, Annemie M. W. J. Schols^C^, Maurice P. Zeegers^AD^

Corresponding author:

Kelly F. J. Stewart, Department of Complex Genetics and Epidemiology, School of Nutrition, and Translational Research in Metabolism (NUTRIM), Maastricht University Medical Centre, Maastricht, The Netherlands
[k.stewart@maastrichtuniversity.nl](mailto:k.stewart@maastrichtuniversity.nl)

Figure 1 Overall meta-analysis of any positive lifestyle change, expressed in proportion of participants changed

Figure 2 Meta-analysis of any positive lifestyle change by consumer type, expressed in proportion of participants changed

Figure 3 Meta-analysis of any positive lifestyle change by price paid, expressed in proportion of participants changed

Figure 4 Overall meta-analysis of improved dietary practices, expressed in proportion of participants changed

Figure 5 Overall meta-analysis of improved exercise practices, expressed in proportion of participants changed

Figure 6 Overall meta-analysis of quitting smoking, expressed in proportion of pre-test smokers who had quit

Figure 7 Overall meta-analysis of change in supplement use, expressed in proportion of participants changed

Figure 8 Meta-analysis of change in supplement use by consumer type, expressed in proportion of participants changed

Figure 9 Meta-analysis of change in supplement use by price paid, expressed in proportion of participants changed

Figure 10 Overall meta-analysis of information-seeking behaviour, expressed in proportion of participants who sought information

Figure 11 Meta-analysis of information-seeking behaviour by customer type, expressed in proportion of participants who sought information

Figure 12 Meta-analysis of information-seeking behaviour by price paid, expressed in proportion of participants who sought information

Figure 13 Overall meta-analysis of sharing with (at least one) health care professional, expressed in proportion of participants who shared

Figure 14 Meta-analysis of sharing with (at least one) health care professional by consumer type, expressed in proportion of participants who shared

Figure 15 Meta-analysis of sharing with (at least one) health care professional by price paid, expressed in proportion of participants who shared

Figure 16 Overall meta-analysis of sharing with a general practitioner, expressed in proportion of participants who shared

Figure 17 Overall meta-analysis of sharing with a genetic specialist, expressed in proportion of participants who shared

Figure 18 Meta-analysis of sharing with a genetic specialist by consumer type, expressed in proportion of participants who shared

Figure 19 Meta-analysis of sharing with a genetic specialist by price paid, expressed in proportion of participants who shared

Figure 20 Overall meta-analysis of sharing with family and/or friends, expressed in proportion of participants who shared

Figure 21 Meta-analysis of sharing with family and/or friends by customer type, expressed in proportion of participants who shared

Figure 22 Meta-analysis of sharing with family and/or friends by price paid, expressed in proportion of participants who shared

Figure 23 Overall meta-analysis of attending preventive checks, expressed in proportion of participants who had preventive checks
